# M^3^ASD: Integrating Multi-Atlas and Multi-Center Data via Multi-View Low-Rank Graph Structure Learning for Autism Spectrum Disorder Diagnosis

**DOI:** 10.3390/brainsci15111136

**Published:** 2025-10-23

**Authors:** Shuo Yang, Zuohao Yin, Yue Ma, Meiling Wang, Shuo Huang, Li Zhang

**Affiliations:** 1 College of Information Science and Technology & Artificial Intelligence, Nanjing Forestry University, Nanjing 210037, China; shuoyang@njfu.edu.cn (S.Y.); yinzuohao@njfu.edu.cn (Z.Y.); lizhang@njfu.edu.cn (L.Z.); 2 School of Clinical Medicine, Jiangsu Health Vocational College, Nanjing 211800, China; mayue@jshvc.edu.cn; 3 School of Computer Science, Nanjing University of Posts and Telecommunications, Nanjing 210023, China; mely@njupt.edu.cn

**Keywords:** autism spectrum disorder, rs-fMRI, multi-center data, multi-atlas fusion, multi-view learning, low-rank representation, graph structure learning, ABIDE

## Abstract

Background: Autism spectrum disorder (ASD) is a highly heterogeneous neurodevelopmental condition for which accurate and automated diagnosis is crucial to enable timely intervention. Resting-state functional magnetic resonance imaging (rs-fMRI) serves as one of the key modalities for diagnosing ASD and elucidating its underlying mechanisms. Numerous existing studies using rs-fMRI data have achieved accurate diagnostic performance. However, these methods often rely on a single brain atlas for constructing brain networks and overlook the data heterogeneity caused by variations in imaging devices, acquisition parameters, and processing pipelines across multiple centers. Methods: To address these limitations, this paper proposes a multi-view, low-rank subspace graph structure learning method to integrate multi-atlas and multi-center data for automated ASD diagnosis, termed M^3^ASD. The proposed framework first constructs functional connectivity matrices from multi-center neuroimaging data using multiple brain atlases. Edge weight filtering is then applied to build multiple brain networks with diverse topological properties, forming several complementary views. Samples from different classes are separately projected into low-rank subspaces within each view to mitigate data heterogeneity. Multi-view consistency regularization is further incorporated to extract more consistent and discriminative features from the low-rank subspaces across views. Results: Experimental results on the ABIDE-I dataset demonstrate that our model achieves an accuracy of 83.21%, outperforming most existing methods and confirming its effectiveness. Conclusions: The proposed method was validated using the publicly available Autism Brain Imaging Data Exchange (ABIDE) dataset. Experimental results demonstrate that the M^3^ASD method not only improves ASD diagnostic accuracy but also identifies common functional brain connections across atlases, thereby enhancing the interpretability of the method.

## 1. Introduction

ASD is a highly heterogeneous neurodevelopmental condition characterized by core symptoms including impaired social interaction and communication, as well as restricted, repetitive patterns of behavior, interests, or activities [1]. Current evidence suggests that ASD arises from the interaction of genetic predispositions and non-genetic environmental factors, leading to widely varying clinical presentations and significant individual heterogeneity. Currently, clinical diagnosis primarily relies on behavioral observation tools—such as the autism diagnostic observation schedule (ADOS)—and questionnaire-based assessments, which are limited by subjectivity and diagnostic delays. According to the Global Burden of Disease Study 2021, the worldwide prevalence of ASD is estimated to be approximately 0.79%, with a 40% increase in screening positivity among young children over the past five years, underscoring the urgent need for more objective and efficient diagnostic tools. With rapid advances in neuroimaging, rs-fMRI has emerged as a promising technique for aiding ASD diagnosis, owing to its ability to reveal abnormal functional connectivity and potential biomarkers within the brain [2]. The growing body of rs-fMRI research offers avenues to overcome the subjectivity inherent in conventional diagnostic approaches, thereby improving diagnostic accuracy and facilitating early identification, timely intervention, and effective treatment of autism.

Early neuroimaging-based studies on ASD primarily relied on feature analysis of functional connectivity networks (FCNs). Conventional approaches often employed Pearson’s correlation coefficient, covariance, or time-series information to quantify temporal correlations in functional activation between brain regions, thereby establishing connectivity between spatially defined regions of interest. However, these methods depend on handcrafted features, which are often subjective, limited in interpretability, reliant on prior knowledge, and inadequate for capturing complex nonlinear interactions. With the widespread adoption of deep learning in medical imaging, significant progress has been made in rs-fMRI-based diagnostic methods for ASD. Initial deep learning applications mainly focused on converting brain network data into grid-like structures for processing. For instance, Yin et al. [3] proposed a semi-supervised autoencoder-based framework for ASD diagnosis, which combines unsupervised autoencoders with supervised classification networks to improve latent feature representation and diagnostic performance compared to traditional hand-engineered FCN features. Heinsfeld et al. [4] employed convolutional neural networks (CNNs) in an end-to-end manner to automatically extract multi-level features from raw imaging data, improving diagnostic accuracy by approximately 12–15%. The intrinsic nature of functional brain networks is non-Euclidean and graph-structured. Converting such data into grid formats—such as by flattening adjacency matrices—can lead to loss of spatial relationships between brain regions. Consequently, graph convolutional networks (GCNs) and transformer models have been introduced to analyze functional brain connectivity, enabling more discriminative representation learning. GCNs are particularly suited to handling graph-structured data directly [5]. Ktena et al. [6] applied GCNs to the ABIDE dataset and directly learning features from functional connectivity graphs for ASD identification, and they have demonstrated significantly better performance compared to traditional machine learning methods. Transformers, known for their ability to model long-range dependencies, have been used to capture dynamic characteristics of rs-fMRI time series. The ST-transformer method proposed by Deng et al. [7] has shown high effectiveness on the ABIDE dataset in handling class imbalance and capturing spatio-temporal features.

Although deep learning has shown promising potential in ASD diagnosis, conventional approaches often rely on a single brain atlas (e.g., AAL or CC200) to construct FCNs, which may lead to insufficient information utilization. Different atlases are based on distinct brain parcellation principles and prior knowledge, potentially capturing complementary information that a single atlas cannot comprehensively cover. To address this limitation, Wang et al. [8] proposed a category-consistent and site-independent multi-view hyperedge-aware hypergraph embedding framework that integrates FCNs constructed from multiple atlases. This method uses hypergraph modeling to capture high-order interactions among brain regions and incorporates specifically designed modules to enhance category discrimination and reduce center-related bias, achieving superior performance in ASD identification on the ABIDE dataset compared to other methods. Similarly, Yu et al. [9] introduced a multi-atlas functional and effective connection attention fusion method, which integrates both functional connectivity and effective connectivity information from multiple atlases using dynamic graph convolutional networks and adaptive self-attention mechanisms, yielding high diagnostic accuracy for ASD. In addition to the limitations of single-atlas approaches, most early studies trained and validated models using single-center data, significantly restricting the generalizability and clinical applicability of these methods. Variations in scanning equipment, acquisition parameters, and participant demographics across imaging centers introduce data heterogeneity, often leading to notable performance degradation when a model trained on one center is applied to another [10]. To mitigate the impact of multi-center data heterogeneity, some researchers have reframed the multi-center ASD diagnosis task as a domain adaptation problem. For instance, Chu et al. [11] incorporated domain adaptation techniques into multi-center analysis by introducing mean absolute error and covariance constraints, effectively reducing inter-center distribution discrepancies. Other studies have proposed low-rank representation-based methods for multi-center ASD identification [12] and low-rank subspace graph convolutional networks [13]. The former employs label propagation to predict unlabeled samples, while the latter uses graph convolutional networks for feature extraction before classification. Wang et al. [14] proposed grouping multi-center data by patient and control categories, using similarity-driven multi-center linear reconstruction to learn latent representations, performing clustering within each group, and applying nested singular value decomposition to reduce center-specific heterogeneity.

To further overcome the limitations of single-atlas information insufficiency and multi-center data heterogeneity, this paper proposes a multi-view, low-rank subspace graph structure learning method to integrate multi-atlas and multi-center data for automated ASD diagnosis, termed M^3^ASD. The proposed framework first constructs functional connectivity matrices from multi-center neuroimaging data using multiple brain atlases. Edge weight filtering is then applied to build multiple brain networks with diverse topological properties, forming several complementary views. Samples from different classes are separately projected into low-rank subspaces within each view to mitigate data heterogeneity. Multi-view consistency regularization is further incorporated to extract more consistent and discriminative features from the low-rank subspaces across views. The proposed method is evaluated on the publicly available ABIDE dataset using multiple data partitioning strategies to demonstrate its effectiveness.

The main contributions of this paper are summarized as follows: (1) We propose a novel multi-view low-rank subspace graph structure learning method, termed M^3^ASD, to integrate multi-atlas and multi-center rs-fMRI data for automated diagnosis of ASD. (2) Our approach overcomes the limitation of relying on a single brain atlas for constructing functional networks. Instead, it leverages multiple brain atlases to build multi-view brain networks with complementary topological properties. (3) To address the heterogeneity inherent in multi-center data, we employ low-rank subspace projection and consistency regularization techniques, effectively mitigating its adverse effects. (4) The proposed M^3^ASD framework is extensively evaluated on the publicly available ABIDE dataset under multiple data partitioning strategies. Experimental results demonstrate its effectiveness in enhancing both the accuracy and generalization capability of ASD diagnosis.

The rest of this paper is organized as follows. In the Materials and Methods section, we describe the datasets used in this study and the proposed method. Then, in the Results section, we present the experimental setup, evaluation index, comparison methods, ablation experiment results, and ASD diagnosis outcomes achieved via different methods. In the Discussion section, we investigate the impact of several key components of the proposed M^3^ASD method. Finally, this paper is concluded in the Conclusions section.

## 2. Materials and Methods

### 2.1. Data Set

#### 2.1.1. Data Source

The ABIDE repository provides a large-scale, publicly available dataset comprising 1112 samples collected from 17 international centers. In this study, a subset of 875 samples was utilized, including 407 individuals with ASD and 468 healthy controls (HC). The age range of the participants was 6–64 years, with a mean age of 17 years. As summarized in Table 1, the data from each center were acquired in clinical settings, reflecting the distribution characteristics of real-world clinical data [15]. The ABIDE initiative aggregates brain imaging data—including resting-state fMRI time series—from individuals with ASD and typically developing children across multiple international research institutions. It aims to facilitate research into the neurophysiology and biomarkers of ASD while also promoting the development of innovative neuroimaging analysis methods. All data in the repository have been anonymized to protect participant privacy, in compliance with relevant ethical guidelines and regulations. ABIDE offers open access to these data, allowing researchers to use them without restriction in order to advance scientific discovery.

#### 2.1.2. Data Preprocessing

To effectively extract biological features from rs-fMRI data, this study employed DPARSF (Version 5.3) (Data Processing Assistant for Resting-State fMRI), a toolbox specifically designed for the automated preprocessing of resting-state fMRI data. The detailed pipeline consists of the following steps: (1) the first 5 time points of each subject’s scan were discarded to eliminate signal instability during the initial phase of acquisition; (2) slice timing correction was applied to ensure temporal alignment across all voxels; (3) head motion artifacts were removed to mitigate spurious correlations caused by subject movement; (4) images were registered to the Montreal Neurological Institute (MNI) standard space and resampled to a resolution of 3 × 3 × 3 mm; (5) bandpass filtering was performed to reduce low-frequency drift and high-frequency physiological noise; (6) Nuisance signals were regressed out; and (7) spatial smoothing was applied using a Gaussian kernel with a full width at half maximum (FWHM) of 4 mm to diminish spatial noise. This comprehensive preprocessing pipeline aims to minimize the influence of non-neurophysiological signals, thereby providing high-quality and standardized data for subsequent analyses.

#### 2.1.3. Atlas Introduction

In this study, three widely-used atlases were employed to extract functional connectivity information between brain regions, as summarized in Table 2. These atlases include the following:

AAL (Automated Anatomical Labeling Atlas): The AAL atlas is a macroscopic anatomically based parcellation scheme. Its key characteristic is the strict adherence to anatomical boundaries, with each region corresponding to a well-defined anatomical label. This atlas divides the brain into 116 distinct regions (90 cortical and 26 subcortical) [16,17].

HO (Harvard-Oxford Atlas): The HO atlas is a probabilistic gray matter segmentation atlas developed through collaborative research at Harvard and Oxford Universities. Rather than providing a binary parcellation, it assigns each voxel a probability of belonging to a specific brain region. Constructed from manually segmented MRI data across multiple subjects, it captures inter-individual anatomical variability more effectively. The HO atlas partitions the brain into 112 regions (96 cortical and 16 subcortical) [17,18].

CC200 (Craddock 200): Proposed by Craddock et al. in 2012 [19], the CC200 atlas is derived using a spectral clustering algorithm applied to resting-state fMRI data. This method groups adjacent voxels with similar time series into functional regions automatically. The resulting atlas comprises 200 regions, enabling the construction of a whole-brain functional connectivity network with 19,900 possible connections (200 × 199/2) [3,19].

### 2.2. Methods

**Figure 1:**
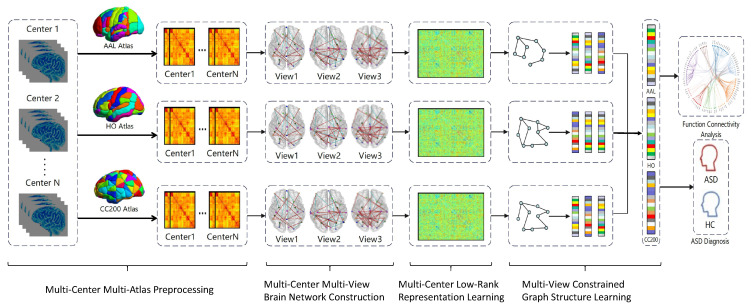
Framework of the M^3^ASD method.

The proposed M^3^ASD method consists of four main modules. The overall framework of the proposed M^3^ASD method is illustrated in Figure 1. (1) Multi-Center Multi-Atlas Preprocessing: rs-fMRI data from multiple centers undergo standardized preprocessing. Different brain atlases (AAL, HO, and CC200) are used to parcellate the brain into regions, from which time series are extracted for each region. (2) Multi-Center Multi-View Brain Network Construction: For the time series derived from each atlas, functional connectivity networks are constructed by filtering connection weights with varying thresholds, resulting in multiple views with distinct topological properties. (3) Multi-Center Low-Rank Representation Learning: views are grouped by diagnostic category and projected into low-rank representation subspaces, enabling the reconstruction of functional connectivity graphs within a low-rank subspace for each subject. (4) Multi-View Constrained Graph Structure Learning (GSL): View consistency regularization is applied to extract feature subnetworks across views. Multi-task graph embedding is then used to learn unified feature representations integrating information from all views. Finally, the multi-view features obtained from the three atlases are fed into a multi-layer perceptron (MLP) for autism diagnosis and interpretable analysis.

#### 2.2.1. Multi-Atlas Multi-Center Preprocessing

Different brain parcellation atlases, based on varying anatomical or functional criteria, offer complementary yet partially overlapping divisions of brain regions and connectivity patterns. In this study, we employed three distinct atlases—AAL, HO, and CC200—to construct functional brain networks, resulting in three separate graph representations. By collectively exploiting the information provided via these multi-atlas networks, we aim to capture latent correlations, complementarities, and discrepancies across atlases. Integrating features derived from different parcellation schemes enables a more holistic and robust representation of brain connectivity, thereby enhancing both the accuracy and generalizability of ASD diagnosis.

Based on the selected brain atlas, the brain is divided into *N* regions of interest (ROIs). Each ROI is considered as a node, $V_{i}$, in the graph G=(V,E), forming the node set $V=\{v_{1},v_{2},\ldots ,v_{n}\}$, and the nodes *V* are pre-ordered. Based on the coordinate definitions of the atlas in the standard structural space, the same ROIs in different individuals have spatial correspondence, ensuring that the constructed graph is spatially aligned and the nodes are ordered, which guarantees comparability across subjects’ brain networks. The edges between nodes, $e_{ij}$, form the edge set $E={\left[e_{ij}\right]}_{N\times N}$, representing structural or functional connections between ROIs. The graph is an undirected weighted graph. $A\in R^{N\times N}$ represents the adjacency matrix of the graph. If there is an edge connection between node $V_{i}$ and $V_{j}$, i.e., $(v_{i},v_{j})\in E$, then $A_{ij}\neq 0$; otherwise, $A_{ij}=0$.

In the graph construction setup, each node $v_{i}$ is associated with a feature vector, $S_{i}$, which characterizes the functional properties of the ROI. The features of all nodes form the node feature matrix, denoted as (where *D* is the feature dimension):

(1) $$S={\left[s_{1},s_{2},\ldots ,s_{n}\right]}^{T}\in R^{N\times D}$$

#### 2.2.2. Multi-View Multi-Center Brain Network Construction

In brain network research, we developed a multi-view multi-center graph construction approach to optimize the analysis of functional connectivity graphs. This method dynamically adjusts connection thresholds to preserve critical inter-regional connectivity while effectively reducing noise interference. For any functional connectivity graph, *X*, of a given subject, an adjustable threshold, α, is used to regulate network sparsity, thereby grouping connections and generating brain networks with distinct topological structures [20]. The node weights in these brain networks are subsequently binarized. A higher value of α (retaining only strong connections) yields a sparse network topology that emphasizes core functional pathways, though it may omit some meaningful weak connections. Conversely, a lower α (retaining more connections) captures more comprehensive connectivity information at the expense of introducing greater noise. The selection of specific values and the number of views is crucial. To ensure a principled and effective multi-view framework, we aimed to integrate brain networks that are not only structurally distinct but also individually informative. Accordingly, we selected three views based on the following rationale: (1) The number of views was set to three to balance model complexity and the diversity of information captured. (2) The thresholds were chosen as α = 0.6, 0.7, and 0.2 because these values were found to produce individual networks with high and complementary discriminative power for ASD diagnosis in our preliminary analysis (see Section 4.2 for detailed justification). This combination allows the model to leverage strong, moderate, and weaker connections in a complementary manner. The mathematical formulation for constructing brain networks under different views is provided below:

(2) $$\varphi_{ij}=\left\{\begin{array}{cc} 1, & b_{ij}>\alpha \\ 0, & |b_{ij}|\leq \alpha \\ -1, & b_{ij}<-\alpha \end{array}\right.$$

Here, $b_{ij}\in X$ represents the connection weight between brain region *i* and brain region *j*. With α = 0.2 taken as an example, the processing of connection weights in the brain network involves the following steps: first, weights greater than 0.2 are set to 1; second, weights between −0.2 and 0.2 are set to 0; and finally, weights less than −0.2 are set to −1.

**Figure 2:**
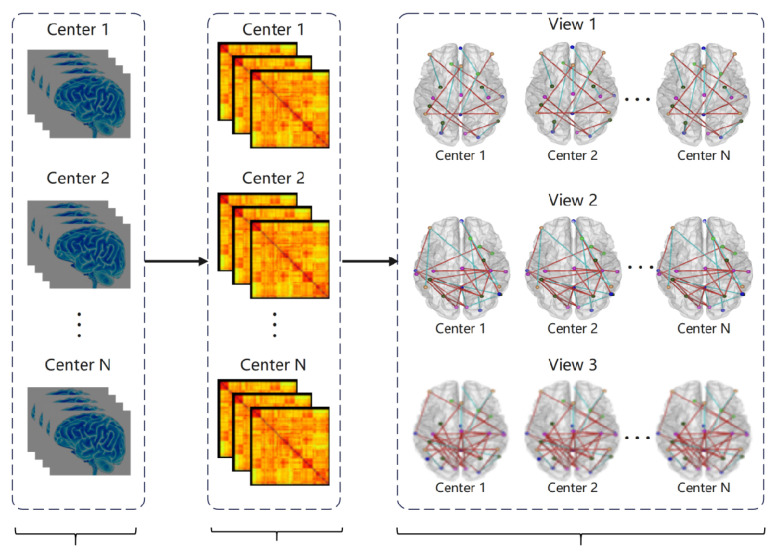
Flowchart of multi-view multi-center brain network construction.

For a single atlas (e.g., AAL), the procedure—as illustrated in Figure 2—begins with standardized preprocessing of fMRI data acquired from multiple scanning centers. Time series data are then extracted based on the chosen atlas. For each subject, a functional connectivity matrix is constructed by computing pairwise correlations between the time series. Finally, three distinct functional brain networks are generated by applying the three thresholds (0.2, 0.6, and 0.7) to the correlation matrix, thereby forming multiple complementary views.

#### 2.2.3. Multi-Center Low-Rank Representation Learning

The core assumption behind using low-rank representation is that data points from different centers but sharing the same class label (e.g., ASD) lie approximately in a common low-dimensional subspace. The center-specific variations and noise are captured by the error matrix $E^{\left(k\right)}$. The nuclear norm minimization on $Z^{\left(k\right)}$ encourages the discovery of this shared subspace, effectively stripping away center-specific biases. The graph Laplacian regularization term further ensures that the geometric structure (i.e., functional connectivity patterns) within the data is preserved in this clean, low-rank subspace. This approach is conceptually distinct from adversarial domain adaptation, as it does not require training a discriminator but instead directly seeks a unified latent structure across centers.

**Figure 3:**
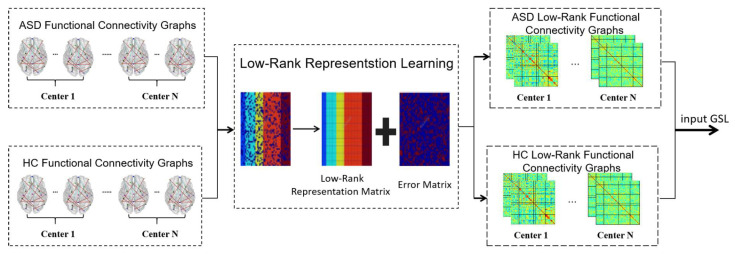
Flowchart of multi-center low-rank representation learning. The module indicated on the right (GSL) is the abbreviation for graph structure learning.

To address the distribution heterogeneity in multi-center neuroimaging data and extract shared feature representations across centers, we introduce a multi-center low-rank representation learning module designed to mitigate inter-center distribution discrepancies. As illustrated in Figure 3, for any given view group, the brain networks are first divided into two groups based on diagnostic labels—ASD and HC (healthy controls). Subsequently, Laplacian-constrained low-rank representation learning [21] is applied to project samples within the same category into a low-rank subspace, thereby reconstructing a low-rank representation of the functional connectivity graph for each subject. Finally, combine ASD and HC from the same view and input them into the subsequent graph structure learning module.

The core algorithm objective function is as follows, where the superscript *k* denotes the *k*-th view; $X^{\left(k\right)}$ represents the functional connectivity graph of ASD or HC; $Z^{\left(k\right)}$ and $E^{\left(k\right)}$ represent the low-rank representation matrix and the error matrix, respectively; $L^{\left(k\right)}$ is the Laplacian matrix; the hyperparameters $\lambda_{1}$ and $\lambda_{2}$ are used to control the weights of the error matrix $E^{\left(k\right)}$ and the Laplacian constraint, respectively.

(3) $$\begin{array}{cc} \min_{Z^{\left(k\right)},E^{\left(k\right)}} & ∥Z^{\left(k\right)}{∥}_{*}+\lambda_{1}{∥E^{\left(k\right)}∥}_{1,2}+\lambda_{2}\;\mathrm{tr}\left(Z^{\left(k\right)}L^{\left(k\right)}{\left(Z^{\left(k\right)}\right)}^{T}\right)\\ s.t. & \;X^{\left(k\right)}=Z^{\left(k\right)}+E^{\left(k\right)},\;k=1,2,\ldots ,n \end{array}$$

The nuclear norm ${∥\;\cdot \;∥}_{*}$ denotes the matrix nuclear norm, defined as the sum of singular values. Its role is to enforce a reduction in the rank of $Z^{\left(k\right)}$.

(4) $${∥Z∥}_{*}=\sum_{i=1}^{r}\sigma_{i}\left(Z\right)$$

The mixed norm ${∥\;\cdot \;∥}_{1,2}$ is the $L_{1,2}$ mixed norm, which computes the $L_{2}$ norm row-wise and then takes the $L_{1}$ sum. It is used to identify and remove center-specific noise while preserving discriminative features.

(5) $${∥E∥}_{1,2}=\sum_{i=1}^{N}\sqrt{\sum_{j=1}^{P}e_{ij}^{2}}$$

where *E* is an N×P matrix, *N* denotes the number of samples, and *P* represents the feature dimension.

The term $\mathrm{tr}(Z^{\left(k\right)}L^{\left(k\right)}{\left(Z^{\left(k\right)}\right)}^{T})$ is the graph structure regularization term, which constrains the feature vectors of adjacent brain regions in the representation space $Z^{\left(k\right)}$ to be as similar as possible, maintaining the biological plausibility of the brain network topology. Introducing the Laplacian matrix constraint in the low-rank representation method ensures that the local adjacency structure of similar samples is preserved in the low-rank representation space. Here, *L* is the unnormalized Laplacian matrix, given as follows:

(6) L=D−A

where *A* is the adjacency matrix, and *D* is the degree matrix.

#### 2.2.4. Graph Structure Learning Based on Multi-View Constraints

**Figure 4:**
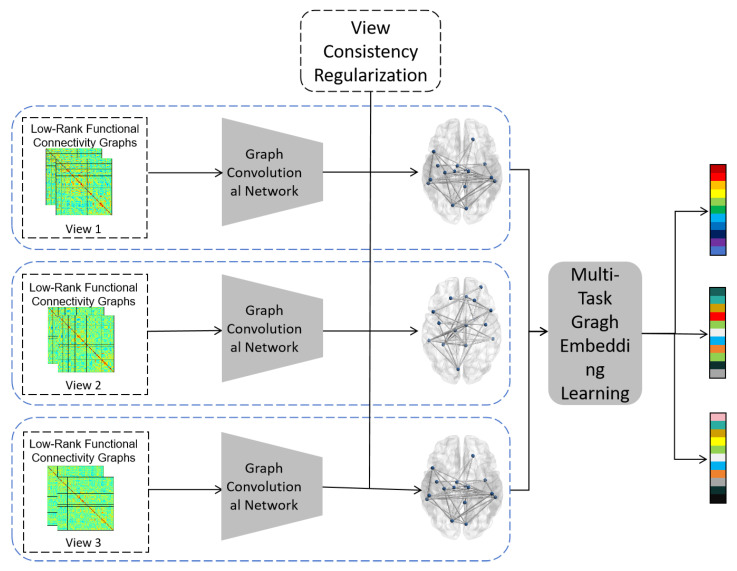
Flowchart of graph structure learning based on multi-view constraints and multi-task graph embedding learning.

FCNs are inherently non-Euclidean data structures (graph structures), where nodes represent brain regions, and edges represent the strength of functional connectivity between regions. Different brain atlases define different brain parcellation schemes, resulting in FCNs with distinct topological properties. To capture critical features across atlas-specific brain networks, further mitigate data heterogeneity across multiple centers, and account for inherent correlations among different views, this paper proposes a multi-view constrained graph structure learning module. As illustrated in Figure 4, the model first learns a more consistent and cleaner brain network under each view through graph structure learning [22], and then acquires feature representations for each sample across different views via multi-task graph embedding, while also capturing latent relationships between views. Furthermore, self-attention mechanisms and view consistency regularization are incorporated into this module to enhance diagnostic performance.

After obtaining the low-rank representation of the functional connectivity graph, this study introduces graph structure learning to coarsen the brain network by aggregating nodes with similar functions into super-nodes, thereby reducing data heterogeneity and noisy connections. The graph structure learning component consists of graph convolutional layers and self-attention-based graph pooling layers. The graph convolutional layers are responsible for iterative node feature refinement, while the self-attention-based graph pooling layers adaptively learn the importance of each node, filter nodes based on their significance, and ultimately extract more discriminative features while reducing the number of learnable parameters.

Self-attention-based graph-pooling methods play a crucial role in graph classification tasks, effectively handling global information extraction and feature learning from graph data, thereby providing strong support for auxiliary diagnosis of brain diseases. Specifically, we employ a standard graph convolutional operation. We first define the augmented adjacency matrix as ${\tilde{A}}^{\left(\ell \right)}=A^{\left(\ell \right)}+I$, where *I* is the identity matrix, which adds self-connections. Its corresponding degree matrix is ${\tilde{D}}^{\left(\ell \right)}$, with its diagonal elements defined as ${\tilde{D}}_{ii}^{\left(\ell \right)}=\sum_{j}{\tilde{A}}_{ij}^{\left(\ell \right)}$. Then, the node feature matrix at the (ℓ+1)-th layer is updated as follows:

(7) $$H^{\left(\ell +1\right)}=\mathrm{ReLU}({\tilde{D}}^{\left(\ell \right)\frac{1}{2}}\tilde{A}{\tilde{D}}^{{\left(\ell \right)}^{-\frac{1}{2}}}H^{\left(\ell \right)}W^{\left(\ell \right)})$$

where $H^{\left(\ell \right)}$ is the node feature graph at the *ℓ*-th layer, ReLU(·) is the activation function, $A^{\left(\ell \right)}$ is the adjacency matrix of the graph at the *ℓ*-th layer, and $A_{i,j}^{\left(\ell \right)}$ represents the connection strength between node *i* and node *j* at the *ℓ*-th layer.

In self-attention based graph pooling, the update formulas for the adjacency matrix $A^{\left(\ell \right)}$ at the *ℓ*-th layer are as follows:

(8) $$S^{\left(\ell +1\right)}=\mathrm{ReLU}\left(\mathrm{SAGPool}\left(\mathrm{GCN}\left(A^{\left(\ell \right)},H^{\left(\ell \right)}\right)\right)\right)$$

(9) $$A^{\left(\ell +1\right)}=S^{(\ell +1)T}A^{\left(\ell \right)}S^{\left(\ell +1\right)}$$

where $S^{\left(\ell +1\right)}$ is the learned cluster assignment matrix at the (ℓ+1)-th layer, and SAGPool(·) denotes the self-attention based graph pooling operation.

Different views constructed with varying thresholds encompass distinct network topological information. However, different views of the same sample inherently possess intrinsic correlations. Therefore, after acquiring network features from different views through graph structure learning, this paper proposes a multi-task graph embedding learning framework to capture the correlations among views. The multi-task graph embedding learning framework consists of two network layers: a shared subnetwork layer and private subnetwork layers. The private subnetwork layers extract unique features from each view, while the shared subnetwork layer learns the correlated features among views and integrates both types of features to obtain comprehensive view representations. For each view, the shared subnetwork layer is defined as follows:

(10) $$Y={\hat{B}}^{\left(k\right)}X_{l}^{\left(k\right)}W_{l}^{\left(k\right)}+X_{l}^{S}$$

where ${\hat{B}}^{\left(k\right)}$ denotes the adjacency matrix of the *k*-th view, $X_{l}^{\left(k\right)}$ represents the subnetwork of the *k*-th view at the *l*-th layer in the shared subnetwork, $X_{l}^{S}$ is the output of the shared subnetwork at the *l*-th layer, and $W_{l}^{\left(k\right)}$ is the learning matrix for the *k*-th view at the *l*-th layer.

To ensure consistency among brain networks from different views, a view consistency regularization term is incorporated into the multi-task graph embedding learning framework. For views $X_{\mathrm{view}(i)}$ and $X_{\mathrm{view}(j)}$, the consistency regularization aims to maximize their similarity, formulated as follows:

(11) $$L_{\mathrm{vc}}=-\sum_{(i,j)\in K}\ln\xi \left(F_{i}F_{j}^{T}\right)$$

where F denotes the correlation matrix obtained from graph structure learning, ξ(·) is the activation function, and *K* represents the set of views in the module.

## 3. Results

### 3.1. Experimental Setup and Index

#### 3.1.1. Experimental Setup

To ensure the reliability of the experimental results, three distinct experimental settings were designed based on sample size and multi-center distribution.

- Setting 1: The five centers with the largest sample sizes in the ABIDE dataset (NYU, UCLA_1, USM, UM_1, PITT) were selected. Each center was sequentially used as the test set, while the samples from the remaining centers formed the training set.
- Setting 2: samples from these five centers were pooled and shuffled to form a unified dataset, which was then split into 80% for training and 20% for testing via cross-validation.
- Setting 3: all 875 extracted samples from the ABIDE dataset were randomly shuffled, with 80% allocated for training and the remaining 20% reserved for testing, also under a cross-validation scheme.

To comprehensively evaluate generalization capability, the three experimental settings each have their specific focus: Setting 1 (Leave-One-Site-Out) primarily aims to test the model’s adaptability to unseen scanning centers, addressing the key challenge of multi-center heterogeneity; Setting 2 (Data Pooling) is used to verify the model’s fundamental ability to learn discriminative features; Setting 3 (Full Dataset) simulates the scenario of applying models trained on large-scale public datasets to clinically sourced data from mixed origins. The heterogeneity of these settings is intentionally designed to validate the model from different perspectives (within-center, between-center, and large-scale out-of-center), and only the comprehensive performance across these results can fully demonstrate the effectiveness and practicality of M^3^ASD.

For the training configuration, the learning rate was set to 0.001 and the maximum number of epochs to 200. The method was developed and evaluated using the PyTorch (Version 2.1.2) deep learning framework on a hardware platform equipped with an NVIDIA RTX 3090 GPU (Santa Clara, CA, USA). The proposed M^3^ASD model required approximately 5 min for a complete training process (200 epochs) under Setting 3. The inference time for a single subject was efficient, averaging around 6 ms. The total number of trainable parameters was 2.15 million, with a peak GPU memory footprint of 4.2 GB during training, demonstrating the practical efficiency of our framework.

#### 3.1.2. Evaluation Index

To evaluate the effectiveness of different methods, this study employs four metrics to assess the performance of the methods, including accuracy (*ACC*), sensitivity (*SEN*), specificity (*SPE*), and the area under the curve (*AUC*) of the receiver operating characteristic (ROC) curve. For these metrics, higher values indicate that the corresponding method can achieve better classification performance. The definitions of *ACC*, *SEN*, and *SPE* are as follows (*TP*, *TN*, *FP*, and *FN* represent true positive, true negative, false positive, and false negative, respectively):

(12) $$\begin{array}{cc} ACC & =\frac{TP+TN}{TP+TN+FP+FN}\;\\ SEN & =\frac{TP}{TP+FN}\;\\ SPE & =\frac{TN}{TN+FP} \end{array}$$

### 3.2. Comparative Methods

To validate the effectiveness of the proposed method, nine representative approaches were selected for comparison, including BrainGNN [22], BNT [23], FBNNetGen [24], MSV-GCN [25], RGTNet [26], GBT [27], AIMAFE [14], 3D-CNN [28], and CNNG [29].

BrainCNN is a GNN variant specifically designed for neuroimaging data, incorporating prior knowledge of brain regions to optimize convolutional kernels. BNT employs self-attention mechanisms to model long-range functional connectivity. FBNNetGen generates synthetic fMRI data to mitigate issues related to small sample sizes and is suitable for cross-dataset transfer learning. MSV-GCN is a multi-view graph convolutional network that reduces bias introduced via single-threshold functional connectivity estimation. RGTNet integrates dynamic functional connectivity analysis, offering greater flexibility than traditional GCNs in capturing complex functional interactions. GBT leverages graph-structured constraints within attention mechanisms to enhance interpretability, making it applicable to multimodal brain network analysis. AIMAFE employs stacked denoising autoencoders to extract discriminative features from multiple atlases for ASD diagnosis. 3D-CNN adopts Poisson disk sampling for optimized feature extraction and uses 3D convolutional networks to integrate multi-atlas information. CNNG combines CNN for spatiotemporal feature extraction from fMRI data with GRU units for final classification.

### 3.3. Experiment Results

#### 3.3.1. Ablation Experiment

The M^3^ASD framework comprises four key modules: multi-center, multi-atlas brain network construction; multi-view, multi-center graph construction; multi-center low-rank representation learning; and multi-view constrained graph structure learning. To evaluate the contribution of each module to the overall diagnostic performance, an ablation study was conducted by systematically excluding individual components.

When the multi-center multi-atlas brain network construction module was ablated, only the AAL atlas was used for subsequent analysis. The removal of the multi-view multi-center graph construction module led to the use of a single view per atlas. If the multi-center low-rank representation learning module was excluded, brain networks derived from different atlases were directly fed into the subsequent graph structure learning phase. Finally, when the multi-view constrained graph structure learning module was removed, the low-rank represented brain networks were classified directly using a softmax classifier.

All ablation experiments were conducted under Experimental Setting 1. Table 3 presents the impact of removing each module on the performance of the M^3^ASD method.

As shown in Table 3, the performance of M^3^ASD decreases significantly when the multi-atlas brain network construction module is removed. This indicates that leveraging multiple atlases allows the model to fully utilize and integrate information from each atlas, thereby effectively enhancing diagnostic performance. Moreover, the performance decline observed when excluding either the multi-center low-rank representation learning module or the multi-view multi-center graph construction module is comparable, suggesting that both modules contribute equally to the overall effectiveness of M^3^ASD. Furthermore, by incorporating the graph structure learning module—which integrates view-consistency constraints—M^3^ASD successfully captures shared features across different views, leading to additional improvements in diagnostic accuracy. We also tested removing combinations of modules, which led to a more severe performance drop than the sum of individual removals, indicating a synergistic effect between the proposed modules.

#### 3.3.2. Comparison with Mainstream Methods

To rigorously evaluate whether the performance improvement of the proposed M^3^ASD over baseline methods is statistically significant, we employed a paired *t*-test. The evaluation metrics obtained from all experimental runs under each setting for each pair of methods were compared. A *p*-value of less than 0.05 was considered to indicate a statistically significant difference.

Under Experimental Setting 1, M^3^ASD outperforms all mainstream methods across all evaluation metrics (*ACC*, *SEN*, *SPE*, and *AUC*), as shown in Table 4. While some baselines achieve competitive results on individual metrics, none surpass M^3^ASD in any category. Overall, M^3^ASD demonstrates the strongest and most balanced performance among all compared methods.

As shown in Table 5, M^3^ASD consistently outperforms all comparison methods across all evaluation metrics under Experimental Setting 2, demonstrating its strong generalization capability in ASD diagnosis. Notably, all methods show improved performance compared to Experimental Setting 1. This enhancement can be attributed to the mixed-center data partitioning scheme used in Setting 2, where samples from five major centers are pooled and randomly split into training and test sets. This approach reduces inter-center heterogeneity in the test set, allowing models to better leverage the training data and achieve more stable performance across all metrics. The superior performance of M^3^ASD under this setting highlights its effectiveness in handling multi-center data while maintaining robust diagnostic accuracy.

Under Experimental Setting 3, which utilizes the full ABIDE dataset of 875 samples from multiple centers, M^3^ASD continues to achieve superior performance across all evaluation metrics, as shown in Table 6. Notably, while the expanded sample size introduces greater inter-center heterogeneity, M^3^ASD maintains robust performance, surpassing all baseline methods in *ACC*, *SEN*, *SPE*, and *AUC*. Compared to Experimental Setting 2, although overall accuracy slightly decreases due to increased data diversity, M^3^ASD still demonstrates a clear advantage over other methods. This result highlights the method’s ability to handle large-scale, multi-center data effectively while mitigating domain shift through low-rank representation learning.

## 4. Discussion

### 4.1. Comparison of the Number of Atlases

This section aims to investigate the impact of the number of atlases on diagnostic performance. For a systematic comparison, all experiments regarding the number of atlases were conducted under Experimental Setting 1, for which the five centers with the largest sample sizes from the ABIDE dataset were selected. Each time, samples from one center were used as the test set, while those from the remaining centers formed the training set.

**Figure 5:**
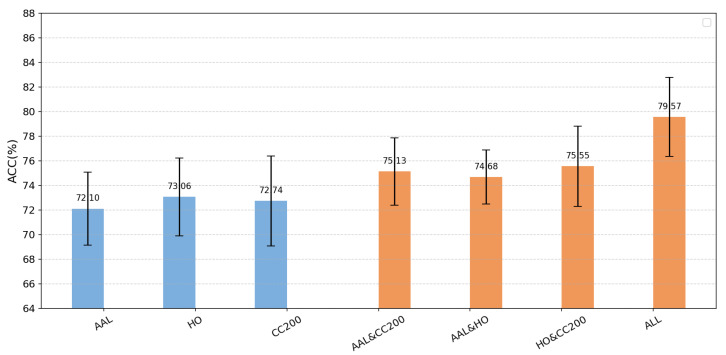
Comparison of accuracy (*ACC*%) between single-atlas and atlas-combination methods. The bar chart displays accuracy values (y-axis, range: 64–88%) for seven atlas configurations (x-axis: AAL, HO, CC200, AAL and CC200, AAL and HO, HO and CC200, ALL). Blue bars represent Single Atlas results (AAL: 72.10%; HO: 73.06%; CC200: 72.74%), while orange bars denote Atlas Combinations (e.g., HO and CC200: 75.55%; ALL: 79.57%). Error bars indicate variability.

We first evaluated the performance using each individual atlas—namely the AAL, CC200, and HO atlases—as input. We then combined the atlases pairwise for comparison and finally integrated all three atlases as input. The corresponding accuracies under these configurations are illustrated in Figure 5. Evidently, the three-atlas input demonstrates a clear advantage over both dual-atlas and single-atlas approaches. Moreover, the accuracy achieved with any two atlases combined exceeded that of any single-atlas method, indicating that multi-atlas integration effectively enhances the accuracy of autism diagnosis [30]. Overall, the multi-atlas approach fully leverages information from each atlas and integrates complementary features, leading to improved overall performance, reduced limitations inherent in single-method analyses, and stronger generalization and diagnostic capability [31].

### 4.2. The Impact of Multi-View Parameters

**Figure 6:**
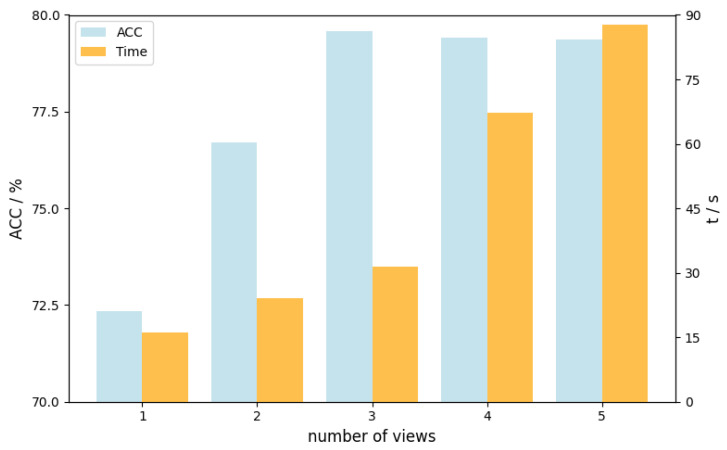
Performance metrics of M^3^ASD under Experimental Setting 1. The dual-axis chart shows classification accuracy (*ACC*%, black line, left y-axis: 70–80%) and computational time efficiency (red line, right y-axis: 0–90 s) versus view quantity (x-axis: 1–5 views). Gray gridlines appear on both axes.

To further investigate the impact of the number of views in the multi-view construction module on method performance, the number of views was incrementally increased from 1 to 5. Under Experimental Setting 1, the *ACC* values and time efficiency of the M^3^ASD method are shown in Figure 6. When the number of views exceeds 3, the performance improvement becomes marginal, while time efficiency decreases significantly. Therefore, this study sets the number of views to three per sample in the M^3^ASD method.

**Figure 7:**
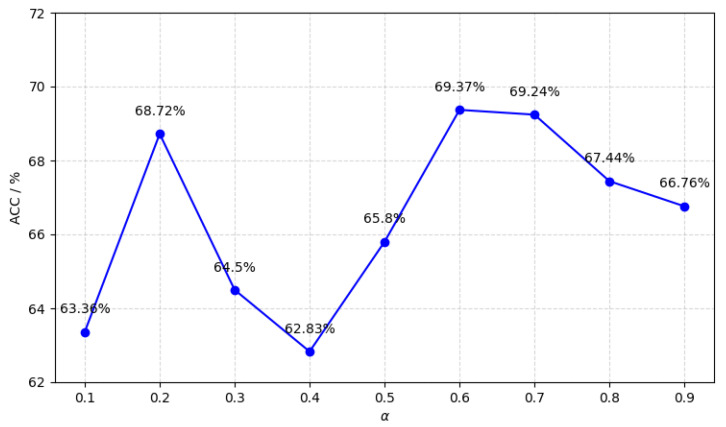
Single-view accuracy versus α parameter values. The line chart displays classification accuracy (*ACC*%, y-axis: 62–72%) across nine α values (x-axis: 0.1–0.9 in 0.1 increments). Blue data points mark measured accuracies (63.36% at α=0.1 to 69.37% at α=0.6), connected by a solid blue trendline. Light gray gridlines appear behind the primary data.

The selection of views also considerably influences method performance. A set of α values, {0.1,0.2,0.3,0.4,0.5,0.6,0.7,0.8,0.9}, was configured, and the accuracy of each single view constructed under different α values was calculated. The results are presented in Figure 7. It was observed that accuracy does not increase linearly with α; instead, the highest accuracy is achieved when α=0.6. The view selection rule in the M^3^ASD method prioritizes views in descending order of their individual accuracy [31]. Thus, when constructing three views, the corresponding α values chosen were 0.6, 0.7, and 0.2.

### 4.3. Important Brain Functional Connections Affecting Multi-Atlas

**Figure 8:**
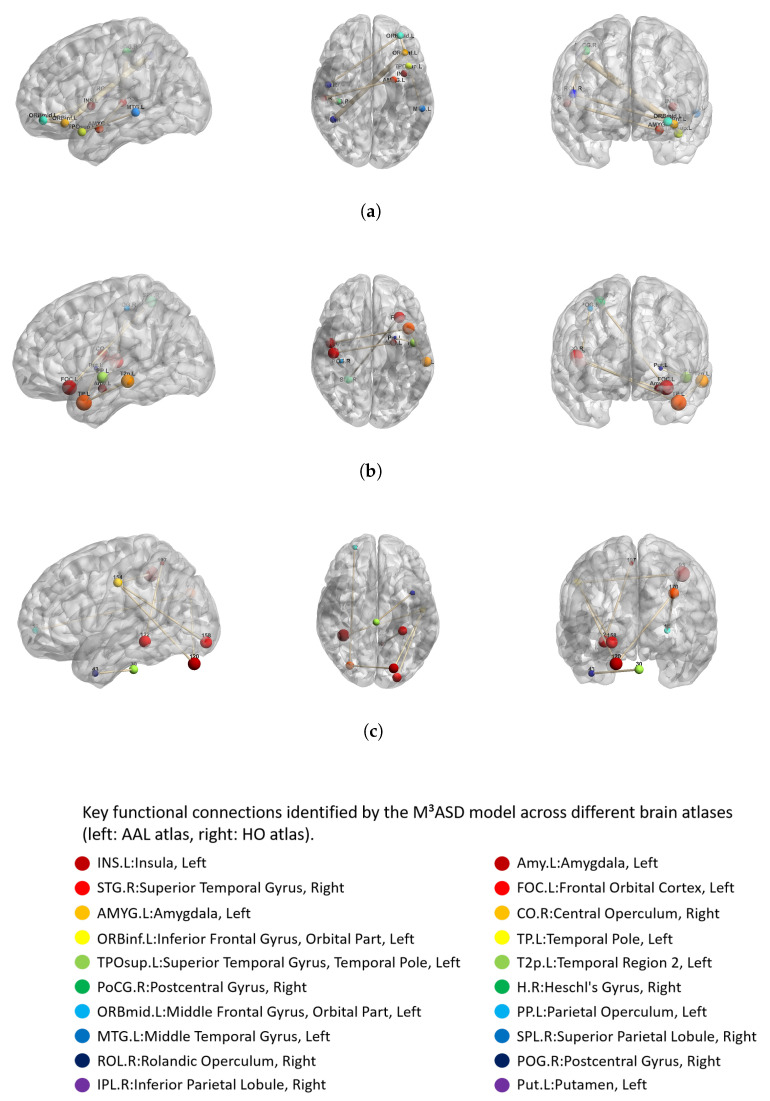
Important brain functional connections in different atlases of ASD symptoms. (**a**) Important brain functional connections affecting ASD in AAL brain atlas. (**b**) Important brain functional connections affecting ASD in HO brain atlas. (**c**) Important brain functional connections affecting ASD in CC200 brain atlas.

This study utilized a multi-center dataset with multiple atlases. After applying low-rank representation, brain network-related weights were learned through a self-attention-based graph convolutional neural network. By analyzing the weights in the functional connectivity weight matrix of each atlas, the top 10 connections with the highest weights were identified, resulting in an important brain functional connectivity graph as shown in Figure 8. This graph reveals brain regions closely associated with ASD pathology and key functional connections between them. As can be observed from the figure, the connection patterns of the AAL atlas and the HO atlas are similar, indicating that although the atlases differ, the brain regions and connections influencing autism are consistent [32]. Through the investigation of significant brain functional connectivity graphs, critical regions [2] potentially strongly linked to autism were identified, including the inferior occipital gyrus, orbital gyrus, insula, superior temporal gyrus [33], amygdala, temporal lobe, and central operculum [34].

To strengthen the interpretability of the key functional connections illustrated in Figure 8, this study conducted systematic statistical validation. Independent samples *t*-tests were performed to compare the functional connectivity strength between the autism spectrum disorder (ASD) and healthy control (HC) groups for the salient connections identified through the M^3^ASD model. The analysis revealed that all critical connections demonstrated statistically significant group differences (all p≤0.05, false discovery rate (FDR) corrected). These results confirm that the connectivity patterns visualized in Figure 8 not only reflect the discriminative basis of the model but also correspond to neuroimaging biomarkers with statistical significance, thereby providing quantitative support for the pathological mechanisms of ASD.

The identified functional connections align well with established models of ASD neurocircuitry. The heightened importance of the connection between the right amygdala and left inferior occipital gyrus [35] is particularly noteworthy. The amygdala is a core hub for emotional processing and social behavior, and its dysfunction is a hallmark of ASD [36]. The inferior occipital gyrus is involved in visual processing. Their aberrant connectivity may underlie difficulties in processing emotionally salient visual stimuli, such as facial expressions, a common challenge in ASD [37]. Furthermore, the consistent identification of the insula and superior temporal gyrus across atlases reinforces their role in ASD. The insula is crucial for interoception and social-emotional awareness, while the superior temporal gyrus is involved in auditory processing and theory of mind. Their disrupted connectivity has been frequently reported in the ASD literature [38]. These findings not only validate the biological plausibility of M^3^ASD but also highlight potential neural pathways for targeted interventions.

### 4.4. Hyperparameter Analysis

To determine the optimal hyperparameter configuration for the M^3^ASD model, we adopted a hierarchical optimization strategy: first identifying the optimal number of graph convolutional layers *k*, followed by a grid search for the regularization parameters $\lambda_{1}$ and $\lambda_{2}$ based on the optimal *k*.

The number of graph convolutional layers *k* determines the depth of neighborhood information aggregation from the functional connectivity networks. A value of *k* that is too small (e.g., k=1) restricts the model’s receptive field, making it difficult to capture long-range dependencies in the brain network, while an excessively large *k* can easily lead to the over-smoothing phenomenon, causing the node features to lose discriminative power. Therefore, we set the search range as k∈{2,3,4,5,6} to balance the model capacity and generalization ability. Under Experimental Setting I, with fixed $\lambda_{1}=0.1$ and $\lambda_{2}=0.01$, we systematically evaluated the impact of different *k* values on the model’s performance. The experimental results indicate that, as *k* increased from 2 to 5, the model accuracy improved steadily, with the accuracy being 62.43±3.42% for *k* = 2, 65.63±2.96% for *k* = 3, 67.59±3.25% for *k* = 4, and reaching its peak of 71.33±2.76% for *k* = 5. However, when *k* was increased to 6, the accuracy decreased to 68.82±3.53%, indicating the occurrence of over-smoothing. This performance trend clearly illustrates the trade-off between network depth and model performance, leading to the determination of k = 5 as the optimal network depth.

The parameter search results, summarized in Table 7, show that the model achieved the highest classification accuracy of 79.57%±3.21% when $\lambda_{1}=0.1$ and $\lambda_{2}=0.05$. This indicates that a moderate $\lambda_{1}$ value effectively filters out noise, while a $\lambda_{2}$ value approximately half its strength well preserves the topological structure of the brain network, with the two parameters working synergistically to achieve optimal performance. A further analysis revealed that, with a fixed $\lambda_{1}$, performance initially increased and then decreased as $\lambda_{2}$ increased. For instance, with $\lambda_{1}=0.1$, accuracy peaked at $\lambda_{2}=0.05$, confirming the effectiveness of the graph structural constraint and the importance of balancing its strength. Moreover, in the vicinity of the optimal combination (e.g., $\lambda_{1}=0.2$, $\lambda_{2}=0.05$ or $\lambda_{1}=0.1$, $\lambda_{2}=0.1$), the model maintained high performance (accuracy >78%), demonstrating that M^3^ASD is relatively insensitive to small perturbations in hyperparameters and possesses good robustness.

In summary, through systematic hyperparameter analysis, we determined the optimal configuration for the M^3^ASD model as k=5, $\lambda_{1}=0.1$, and $\lambda_{2}=0.05$. This configuration achieves an optimal balance between model expressive power, structure preservation, and noise suppression, providing a reliable parameter foundation for subsequent experiments.

### 4.5. Limitation and Future Work

#### 4.5.1. Limitation

Despite the encouraging results, this study is subject to several limitations. Firstly, the validation was solely reliant on the ABIDE-I dataset. Although our method is designed to handle multi-center heterogeneity, its generalizability to completely independent cohorts (e.g., ABIDE-II, EU-AIMS) remains to be further verified. The absence of external validation constitutes a limitation of the current work. Secondly, while the multi-center and multi-atlas design is a core strength of this study, it inherently limits the sample size available within each individual center. Consequently, conducting statistically meaningful subgroup analyses (e.g., based on specific gender categories, symptom severity scores, or IQ levels) would result in extremely small and underpowered subgroups per center, potentially leading to unreliable conclusions. Therefore, our current analysis focused on validating the overall framework’s efficacy across centers.

#### 4.5.2. Future Direction

Future research will prioritize the following directions to address these limitations: (1) Applying M^3^ASD to larger, independent multi-center cohorts to facilitate both robust external validation and meaningful subgroup analyses. (2) Collaborating with multiple institutions to aggregate larger sample sizes within specific demographic or clinical subgroups, enabling the development of more personalized diagnostic models. (3) Exploring the integration of additional neuroimaging modalities to provide a more comprehensive characterization of ASD.

While this study establishes the utility of M^3^ASD for multi-center, multi-atlas static functional connectivity analysis, several exciting avenues emerge for extending its impact. The inherent temporal dynamics of ASD present a clear next step: integrating models like long short-term memory (LSTM) networks to analyze longitudinal rs-fMRI data could significantly enhance pattern recognition of disease progression.

Beyond temporal analysis, the multi-view architecture of M^3^ASD is inherently extensible to other data modalities. Future work could incorporate electrophysiological data (e.g., EEG/EMG) from wearable sensors or leverage advances in photonic sensing technology [39], moving towards a comprehensive multi-modal diagnostic system. The core methodology of M^3^ASD is also not specific to ASD. It holds considerable potential for application to other neurological disorders characterized by aberrant network patterns, such as ADHD or schizophrenia.

The ultimate translational goal is the development of systems for continuous patient monitoring and neurorehabilitation. Achieving this requires addressing challenges such as model lightweighting and integration with portable systems [40] to enable real-time clinical decision support. To ensure reliability in heterogeneous clinical populations, future iterations must incorporate uncertainty quantification (e.g., via Bayesian deep learning) to provide predictive confidence scores, which is crucial for clinical trust.

## 5. Conclusions

In this study, we developed the M^3^ASD method for automated ASD diagnosis using rs-fMRI data. Within the M^3^ASD framework, we utilized 875 multi-center samples from the ABIDE dataset. For each subject, multiple brain atlases were first applied for ROI parcellation, followed by the construction of multi-scale FCNs under a multi-view setting. The brain networks then underwent low-rank representation learning to mitigate heterogeneity across data centers. Finally, we employed self-attention mechanisms combined with prior subnetwork constraints for graph structural learning, enabling effective feature fusion and classification.

The proposed method was evaluated under multiple experimental setups and compared against several state-of-the-art approaches. Results demonstrate its effectiveness in both FCN feature learning and ASD diagnosis. In future work, we aim to develop and validate stage-specific diagnostic models tailored to distinct developmental periods—such as preschool, school-age, adolescence, and adulthood—to significantly enhance diagnostic stability and accuracy within targeted populations, particularly those requiring early and sensitive detection [41].

## Figures and Tables

**Table 1:** Statistics of ABIDE dataset information.

CENTER_ID	ASD		HC	
	**Sex (M/F)**	**Age**	**Sex (M/F)**	**Age**
CALTECH	15/4	27.44 ± 10.30	14/4	28.02 ± 10.89
CMU	3/0	30.33 ± 8.50	1/1	25.50 ± 6.36
KKI	9/3	9.56 ± 1.40	20/6	10.13 ± 1.11
LEUVEN_1	14/0	21.86 ± 4.11	15/0	23.27 ± 2.91
LEUVEN_2	11/2	13.81 ± 1.06	14/5	14.22 ± 1.45
MAX_MUN	15/3	30.44 ± 13.99	23/1	25.92 ± 8.32
NYU	63/9	14.96 ± 7.13	72/26	15.67 ± 6.22
OHSU	12/0	11.43 ± 2.18	11/0	10.37 ± 1.10
OLIN	11/3	16.79 ± 3.77	9/2	17.55 ± 3.17
PITT	18/4	19.35 ± 7.52	20/3	19.13 ± 6.32
SBL	14/0	35.29 ± 10.76	12/0	34.42 ± 6.04
SDSU	12/0	15.05 ± 1.67	15/6	14.32 ± 1.89
STANFORD	13/4	10.15 ± 1.65	15/4	9.89 ± 1.62
TRINITY	21/0	17.01 ± 3.12	23/0	17.48 ± 3.66
UCLA_1	26/2	13.62 ± 2.69	23/4	13.52 ± 1.95
UCLA_2	8/0	12.35 ± 2.06	10/2	12.40 ± 1.03
UM_1	28/8	13.44 ± 2.41	26/14	14.53 ± 3.02
UM_2	11/1	15.05 ± 1.49	18/1	16.94 ± 4.12
USM	38/0	24.60 ± 8.57	23/0	22.33 ± 7.87
YALE	15/7	13.01 ± 3.10	18/7	12.63 ± 2.82
All Centers	357/50	17.70 ± 8.94	382/86	16.87 ± 7.37

**Table 2:** Brain region division of each atlas.

Atlas Name	Total Number of Regions	Number of Cortical Regions	Number of Subcortical Regions
AAL	116	90	25
HO	112	96	16
CC200	200	N/A	N/A

**Table 3:** Results of the ablation experiment.

Multi-Atlas	Multi-Views	Low-Rank	GSL	Performance Metrics			
				***ACC*** **(%)**	***SEN*** **(%)**	***SPE*** **(%)**	***AUC*** **(%)**
✓	✓			62.37±4.22	63.41±3.77	60.65±3.48	61.98±3.51
✓		✓		61.84±3.66	62.76±4.13	60.32±4.41	62.21±3.79
✓			✓	64.23±3.56	65.88±4.43	62.38±4.20	63.96±3.74
	✓	✓		56.78±4.69	60.21±4.36	54.47±3.80	57.23±4.64
	✓		✓	58.49±4.52	60.87±4.38	56.24±3.77	59.34±4.26
		✓	✓	55.42±5.37	56.71±4.86	55.19±4.36	54.68±4.60
	✓	✓	✓	67.85±4.05	70.88±3.77	65.58±3.56	67.91±3.32
✓		✓	✓	71.29±3.22	73.30±3.01	75.03±2.95	71.84±3.17
✓	✓		✓	71.73±2.78	74.52±3.21	75.86±3.11	72.51±2.90
✓	✓	✓		74.87±4.11	75.66±3.92	72.88±3.78	74.03±4.02
✓	✓	✓	✓	79.57±3.21	81.21±4.55	76.54±4.13	79.07±3.53

**Table 4:** Results of various methods on experimental Setting 1.

Methods	*ACC* (%)	*SEN* (%)	*SPE* (%)	*AUC* (%)
BrainGNN	59.08±2.71	69.45±1.86	50.08±2.47	61.71±1.46
BNT	65.48±3.53	65.24±3.53	65.70±3.44	70.33±3.78
FBNetGen	62.19±6.51	73.23±6.98	52.43±7.64	61.30±4.13
MSV-GCN	64.28±4.12	74.50±2.77	54.93±4.47	61.83±3.53
RGTNet	70.11±2.08	70.09±1.63	70.19±1.76	69.17±1.76
GBT	66.38±1.92	66.46±1.66	66.22±2.30	74.13±1.42
AIMAFE	70.22±2.71	67.19±5.59	65.41±3.98	71.59±5.66
3D-CNN	70.65±2.97	67.86±6.41	73.16±6.32	73.48±5.67
CNNG	73.21±1.65	70.13±3.51	76.16±4.68	74.27±3.54
M^3^ASD	79.57±3.21 *	81.21±4.55 *	76.54±4.13 *	79.07±3.53 *

* The improvement of M^3^ASD over all other methods is statistically significant (p<0.05) based on a paired *t*-test.

**Table 5:** Results of various methods on Experimental Setting 2.

Methods	*ACC* (%)	*SEN* (%)	*SPE* (%)	*AUC* (%)
BrainGNN	63.43±2.69	74.95±1.87	53.41±2.42	66.35±1.46
BNT	68.80±3.61	68.46±3.57	69.10±3.48	73.84±3.85
FBNetGen	71.48±6.61	82.05±9.09	58.53±7.73	68.36±4.30
MSV-GCN	67.52±4.34	81.28±2.88	59.85±4.42	65.76±3.27
RGTNet	76.30±2.19	76.37±1.62	76.24±1.81	75.11±1.56
GBT	69.52±2.06	69.70±1.43	69.37±2.20	77.78±1.37
AIMAFE	81.00±5.22	90.01±5.04	74.25±5.59	89.61±5.66
3D-CNN	78.26±3.00	73.48±3.39	79.80±3.57	80.59±5.78
CNNG	76.64±3.57	77.57±3.52	81.74±4.75	78.91±3.59
M^3^ASD	87.22±4.13 *	85.38±5.43 *	82.73±4.98 *	87.64±4.11 *

* The improvement of M^3^ASD over all other methods is statistically significant (p<0.05) based on a paired *t*-test.

**Table 6:** Results of various methods on Experimental Setting 3.

Methods	*ACC* (%)	*SEN* (%)	*SPE* (%)	*AUC* (%)
BrainGNN	60.41±2.61	71.38±1.82	50.87±2.35	63.19±1.42
BNT	66.80±3.54	66.47±3.50	67.09±3.41	71.69±3.77
FBNetGen	66.80±6.36	76.68±8.74	54.70±7.43	63.89±4.13
MSV-GCN	64.92±4.21	78.15±2.80	57.55±4.29	63.23±3.17
RGTNet	71.98±2.11	72.05±1.56	71.92±1.74	70.86±1.50
GBT	68.16±2.02	68.33±1.40	68.01±2.16	76.25±1.34
AIMAFE	75.00±4.97	83.34±4.80	68.75±5.32	83.9±5.39
3D-CNN	74.53±2.91	69.98±3.29	76.00±3.47	76.75±5.61
CNNG	72.47±3.50	74.34±3.45	79.36±4.66	73.70±3.52
M^3^ASD	83.21±3.34 *	81.43±4.39 *	78.92±4.03 *	83.61±3.32 *

* The improvement of M^3^ASD over all other methods is statistically significant (p<0.05) based on a paired *t*-test.

**Table 7:** Comparison of hyperparameter results.

$\lambda_{2}$/$\lambda_{1}$	0.01	0.02	0.05	0.1	0.2
0.005	63.34±3.13	65.71±2.88	66.34±2.23	69.49±2.41	68.72±3.32
0.01	65.77±2.65	67.91±3.11	69.61±2.47	71.33±2.76	68.13±3.07
0.02	71.78±2.71	73.40±2.88	73.98±3.11	74.23±2.45	71.43±2.94
0.05	74.86±2.98	75.67±2.42	77.32±3.23	79.57±3.21	78.07±3.16
0.1	75.42±2.70	75.17±3.19	77.39±2.55	78.36±2.72	76.73±3.22

## Data Availability

The original neuroimaging data presented in this study are openly available in the ABIDE I dataset at https://fcon_1000.projects.nitrc.org/indi/abide/abide_I.html, accessed on 17 July 2025. The source code and implementation of the M^3^ASD framework are available at the GitHub repository: https://github.com/shuoyang031102/, accessed on 15 July 2025.
